# Magnetic particle mapping using magnetoelectric sensors as an imaging modality

**DOI:** 10.1038/s41598-018-38451-0

**Published:** 2019-02-14

**Authors:** Ron-Marco Friedrich, Sebastian Zabel, Andreas Galka, Nils Lukat, Jan-Martin Wagner, Christine Kirchhof, Eckhard Quandt, Jeffrey McCord, Christine Selhuber-Unkel, Michael Siniatchkin, Franz Faupel

**Affiliations:** 1 Christian-Albrechts-Universität zu Kiel, Institute for Materials Science, Chair for Multicomponent Materials, Kiel, 24143 Germany; 2 Christian-Albrechts-Universität zu Kiel, Institute for Medical Psychology and Medical Sociology, Kiel, 24105 Germany; 3 Christian-Albrechts-Universität zu Kiel, Institute for Materials Science, Biocompatible Nanomaterials, Kiel, 24143 Germany; 4 Christian-Albrechts-Universität zu Kiel, Institute for Materials Science, Functional Nanomaterials, Kiel, 24143 Germany; 5 Christian-Albrechts-Universität zu Kiel, Institute for Materials Science, Chair of Inorganic Functional Materials, Kiel, 24143 Germany; 6 Christian-Albrechts-Universität zu Kiel, Institute for Materials Science, Nanoscale Magnetic Materials and Magnetic Domains, Kiel, 24143 Germany

## Abstract

Magnetic nanoparticles (MNPs) are a hot topic in the field of medical life sciences, as they are highly relevant in diagnostic applications. In this regard, a large variety of novel imaging methods for MNP in biological systems have been invented. In this proof-of-concept study, a new and novel technique is explored, called Magnetic Particle Mapping (MPM), using resonant magnetoelectric (ME) sensors for the detection of MNPs that could prove to be a cheap and efficient way to localize the magnetic nanoparticles. The simple and straightforward setup and measurement procedure includes the detection of higher harmonic excitations of MNP ensembles. We show the feasibility of this approach by building a measurement setup particularly suited to exploit the inherent sensor properties. We measure the magnetic response from 2D MNP distributions and reconstruct the distribution by solving the inverse problem. Furthermore, biological samples with magnetically labeled cells were measured and reconstruction of the distribution was compared with light microscope images. Measurement results suggest that the approach presented here is promising for MNP localization.

## Introduction

Magnetic nanoparticles (MNP) are used in a variety of applications in the field of medical life sciences^1^. Due to their biocompatibility and magnetic properties, possible applications include the diagnostics and treatment of cancer^2–5^ and the labeling and imaging/tracking of cells^1,6,7^. A great benefit of imaging MNP distributons using advanced magnetic imaging systems is that the sample can be investigated in a non-destructive and non-invasive fashion. The general interest in the imaging of MNP distributions gave rise to a variety of imaging systems using and detecting the magnetic responses and influences of the MNPs. The most prevalent imaging systems for the detection of MNPs as an imaging modality includes magnetic resonance imaging (MRI)^1,7^, magnetic particle imaging (MPI)^8,9^ and magnetorelaxometry imaging (MRX)^10^. MRI detects the effect of the nanoparticles indirectly through their influence on the relaxation time of hydrogen, replacing radioactive contrast agents. MPI employs the nonlinear magnetic behavior of the particles and uses large field gradients to locally detect MNPs. MRX uses the relaxation time of the MNPs after the magnetic moments had been aligned by an external field. Other imaging systems for MNPs include, but not exclusively, the usage of ultrasound^11,12^, electron paramagnetic resonance^13,14^ or AC-susceptibility^15^. However, advanced magnetic imaging systems such as MPI or MRI are highly sophisticated systems and therefore expensive. MRX often uses superconducting quantum interference devices (SQUIDs) to detect the relaxation of MNPs, but also fluxgate magnetometers have been used^16^. Detecting magnetic responses from MNPs can therefore be achieved via a multitude of sensors. Still, the usage of small, cheap and sensitive sensors in imaging arrays could greatly enhance detection and imaging of MNPs.

In this proof-of-concept study we propose a novel strategy, called magnetic particle mapping (MPM), to locate MNPs by detecting their nonlinear magnetic response to a monofrequent magnetic excitation field, using a single resonant magnetoelectric (ME) sensor of dimensions 3×1 mm^2^ in a scanning setup. ME sensors are composite materials that convert magnetic fields into a voltage via a mechanical coupling of a magnetostrictive and a piezoelectric phase. Because these ME sensors are fabricated using MEMS technology, mass produced sensors could be used for magnetic field imaging arrays for a fast and cheap localization of magnetic particle distributions. Recently, ME sensors have been proposed to be used in clinical interventions by detecting MNPs^17^.

The ME sensors used in this study are freestanding cantilevers exhibiting mechanical resonances, which are able to amplify magnetic field signals. The sensors exhibit a low limit of detection (LOD) in the order of 100 pT/(Hz)^0.5^at 10 Hz using modulation techniques^18^ and even better LOD in direct detection at the mechanical resonance frequency of the sensor. The sensitivity, called the magnetoelectric coefficient *α*_ME_, is comprised of its magnetostrictive response λ to the magnetic field *H* and the piezoelectric response *V*_ME_ to the mechanical stress σ, which are mechanically coupled^19^:

1 $${\alpha }_{{\rm{ME}}}=\frac{\partial {V}_{{\rm{ME}}}}{\partial \sigma }\frac{\partial \sigma }{\partial \lambda }\frac{\partial \lambda }{\partial H}$$

In this paper, we use an ME sensor to measure the magnetic fields of MNPs by measuring higher harmonic excitations from a monofrequent magnetic excitation. We utilize the special properties of the sensor to amplify the particle signals and decouple it from the excitation signal. We model the imaging system under these considerations and implement a measurement setup using a single sensor. We then detect and map the magnetic field of spatial distributions of MNPs in 2D and solve the inverse problem using the model to reconstruct the particle distribution. We further use the method to detect the location of magnetically labeled cells, as an example of a biological application, where we confirm the reconstructed cell distribution from the MPM method by light microscopy images.

## Experimental Setup

**Figure 1:**
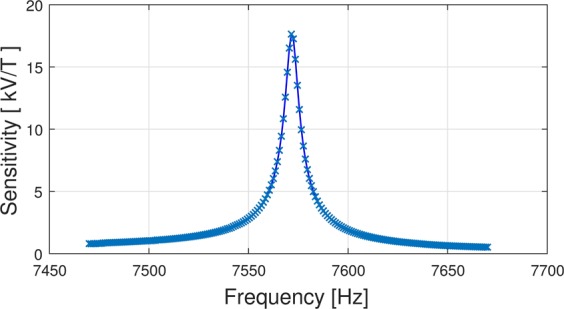
Frequency response of the sensitivity of the sensor.

The ME sensor which we use in this paper, exhibits a strong bandpass behavior with a bandwidth of about 10 Hz and a mechanical resonance at about 7.55 kHz. The sensor is exchange biased to eliminate the need of an external magnetic bias field and to operate at large sensitivities. The sensitivity of the ME sensor also depends strongly on the applied frequency of the magnetic field, being largest at the resonance frequency (see Fig. 1). In our case, the maximum sensitivity is 18 kV/T, using a charge amplifier (AD745). The fabrication process and the materials used are explained at the end of this paper and noise spectra of the sensor can be found in the Supplementary Material.

**Figure 2:**
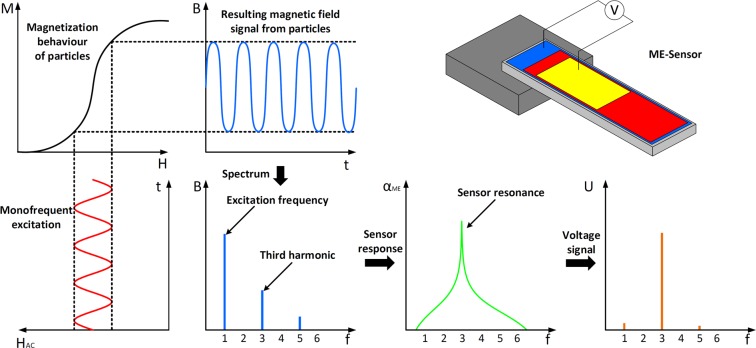
Generation of higher harmonic excitations due to nonlinear magnetic behavior and subsequent bandpass filtering because of the ME sensor. The top right shows a principle shape of the sensor with an electrode to measure the voltage signal, the red layer denotes the piezoelectric material and the blue one denotes the magnetostrictive layer (sensor schematic recreated from^29^).

The measurement approach we use is shown in Fig. 2. We exploit the sharp resonance of the sensor and the nonlinear magnetic behavior of the MNP to decouple the particle signal from the excitation. Because MNP ensembles exhibit a nonlinear magnetization behavior, a monofrequent external magnetic field leads to the generation of higher harmonic excitations for the magnetization of MNP ensembles. By exciting the MNPs in an alternating magnetic field with a frequency equal to 1/*n* of the mechanical resonance of the sensor, we can measure the *n*^th^ harmonic excitation of the MNPs at the mechanical resonance. Because the first harmonic excitation is superimposed by the external excitation field, we cannot distinguish between these fields and generally the excitation field is several orders of magnitude larger than the particle signal, especially for small magnetic contents. We are thus limited to the harmonic excitations with *n* > 1. The 2^nd^ harmonic excitation is in this case nonexistent, because we operate around the origin of the magnetization curve, which is antisymmetric. We thus choose the 3^rd^ harmonic excitation for the measurements, because it gives the largest signal of the remaining harmonics.

We further need to ensure that the influence of nonlinearity in the excitation system does not superimpose with the particle signal. For this, we employ the directional sensitivity of the sensor. The sensor measures a single projection of the magnetic field onto the axis perpendicular to the in-plane magnetization^20^. Therefore, if we excite perpendicular to the plane of the sensor, we can further attenuate influences of the excitation field in the measurements, because the demagnetization factor in that direction is basically equal to one.

Using these principles, small fields can be measured such that the particle signal can still be resolved without falling out of the dynamic range of the A/D-converter used for the readout of the sensor signal. Experiments have been carried out using an ME sensor to map the magnetic field of MNPs distributed in a plane. We detect the third harmonic excitation of the MNPs using an excitation field amplitude of 10 mT*μ*_0_^−1^.

## Modeling

We model the forward problem as a space invariant linear system, meaning that the fields of the MNPs superimpose without affecting each other and, in this case, that all MNPs have the same orientation of their magnetic moment. On this basis, we formulate the inverse problem and solve the inverse problem in the least square sense with an L2 penalty term, i.e. using the Euclidean norm, to regularize the solution vector. We also impose a positivity constraint to account for the semi-positive particle concentration. Modeling of the measurement approach needs to be examined in two ways: How does the measurable magnetic field depend on the particle distribution and how is the nonlinear magnetic behavior translated into a higher harmonic excitation?

To answer the latter, we assume that the MNPs behave superparamagnetic, such that the magnetization behavior can be described by the Langevin function, and that the particles act as magnetic point dipoles. In thermodynamic equilibrium, the mean magnetic moment *μ* is given by a fraction of a maximum magnetic moment *μ*_max_:

2 $$\mu ={\mu }_{{\rm{\max }}}L(\xi )$$

Here, *L*(*ξ*) is the Langevin function, which describes the extent of magnetization of an ensemble of magnetic particles for a certain magnetic field. It is given by

3a $$L(\xi )=\,\coth (\xi )-\frac{1}{\xi },\,{\rm{with}}$$

3b $$\xi =\frac{|{\mu }_{{\rm{\max }}}|{\mu }_{0}|\overrightarrow{H}|}{{k}_{B}T}$$

As can be seen from Eq. (3b), the parameter *ξ*, also known as the Langevin parameter, is related to the maximum strength of the magnetic moment and the applied magnetic field. Here, *μ*_0_ is the vacuum permeability, *k*_B_ is the Boltzmann constant, *T* the absolute temperature and $\overrightarrow{H}$ the magnetic field. The function describes the nonlinear magnetic behavior of an ensemble of particles. It is important to note that the Langevin function describes the equilibrium and that the emerging magnetic moment of an ensemble is only defined in the direction of the applied magnetic field.

To answer how the particle distribution contributes to the measurable magnetic field we assume that each particle will act as a single large magnetic dipole with a certain magnetic moment $\overrightarrow{\mu }$. Associated with such a moment is also a magnetic dipole field ${\overrightarrow{B}}_{{\rm{D}}}$. By a particle, a magnetic field is generated that can be described by the following equation:

4 $${\overrightarrow{B}}_{{\rm{D}}}=\frac{{\mu }_{0}}{4\pi {r}^{5}}[3\overrightarrow{r}(\overrightarrow{\mu }\cdot \overrightarrow{r})-\overrightarrow{\mu }{r}^{2}]=\frac{{\mu }_{0}|\mu |}{4\pi {r}^{5}}[3\overrightarrow{r}(\hat{\mu }\cdot \overrightarrow{r})-\hat{\mu }{r}^{2}]$$

The vector of the dipole moment can be written as $\overrightarrow{\mu }=|\mu |\hat{\mu }$, where $\hat{\mu }$ is the unit vector of the dipole moment. The vector $\overrightarrow{r}$ is the distance from the magnetic moment. The direction of the magnetic moment can be changed with the applied magnetic field direction. Assuming a monodisperse particle size distribution and, to accommodate for the fact that the Langevin function describes a statistical behavior, a large amount of particles in the sample, we can rewrite the magnetic moment with the Langevin function to describe the field of the magnetic moment of a particle as a function of position and Langevin parameter:

5 $${\overrightarrow{B}}_{{\rm{D}}}(\overrightarrow{r},\xi )=\frac{{\mu }_{0}|{\mu }_{{\rm{\max }}}|}{4\pi |\overrightarrow{H}|{r}^{5}}[3\overrightarrow{r}(\overrightarrow{H}\cdot \overrightarrow{r})-\overrightarrow{H}{r}^{2}](\coth (\xi )-\frac{1}{\xi })$$

Now simplifying and introducing the amplitude of the magnetic field ${\overrightarrow{B}}_{{\rm{D}},0}$ for every position in space yields:

6 $${\overrightarrow{B}}_{{\rm{D}}}(\overrightarrow{r},\xi )={\overrightarrow{B}}_{{\rm{D}},0}\cdot (\coth (\xi )-\frac{1}{\xi }),$$

7 $${\overrightarrow{B}}_{{\rm{D}},0}=\frac{{\mu }_{0}|{\mu }_{{\rm{\max }}}|}{4\pi |\overrightarrow{H}|{r}^{5}}[3\overrightarrow{r}(\overrightarrow{H}\cdot \overrightarrow{r})-\overrightarrow{H}{r}^{2}].$$

We can see that the amplitude is constant with respect to *ξ* and thus a fraction of the spatial amplitude will be measured at the resonance frequency, depending on the nonlinearity of the Langevin function and the maximum magnetic field amplitude. We also see that ${\overrightarrow{B}}_{{\rm{D}},0}$ scales linearly with *μ*_max_, meaning that any signal strength will scale linearly with the amount of magnetic moment.

**Figure 3:**
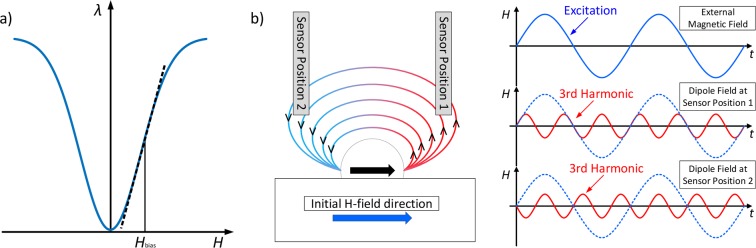
(**a**) Schematic magnetostriction curve. Internal bias field to gain the largest sensitivity of the sensor. The origin of the graph is located at *H*=*H*_bias_, such that, at zero external field, the sensor operates at the largest slope. The sensitivity of the sensor is directly proportional to the slope of the magnetostriction curve. (**b**) Depending on the sensor position, a phase difference of *π* occurs in the third harmonic excitation.

The magnetic particles will be exposed to an alternating magnetic field that has 1/3 of the frequency of the resonance frequency of the sensor. The third harmonic excitation due to the nonlinear behavior of the MNPs will therefore be measured. Here we use an exchange biased sensor, *i*.*e*. we are operating at the largest slope of the magnetostriction curve [see Fig. 3a)]. For more details on the exchange biasing see ref.^21^. Because it is related to the sensitivity [see Eq. (1)], we can linearize the curve. This can be used to measure a phase difference with respect to the excitation signal to determine the sign of the magnetic field. This is illustrated in Fig. 3b). The magnetic field will be applied such that there is a global excitation of the whole sample, leading to all magnetic moments pointing in the same direction. The forward modeling can then be described by a convolution with the field of the magnetic point dipole, because this gives us the superposition of all magnetic field sources. This is sufficient because the magnetic particles are very small. Since we are talking about the distribution of magnetic particles in a plane, the forward modeling is analogous to, *e*.*g*., the blurring process in a defocused camera. That way, the dipole field corresponds to a Point-Spread-Function (PSF), referred to as $\Psi$ in this paper. Thus, the relationship that describes the magnetic field *y* resulting from a particle distribution *x* is given by:

8 $$y=x\ast \Psi =\underset{A}{\overset{\,}{\iint }}x({\tilde{a}}_{1},{\tilde{a}}_{2})\Psi ({a}_{1}-{\tilde{a}}_{1},{a}_{2}-{\tilde{a}}_{2}){\rm{d}}{\tilde{a}}_{1}{\rm{d}}{\tilde{a}}_{2}$$

**Figure 4:**
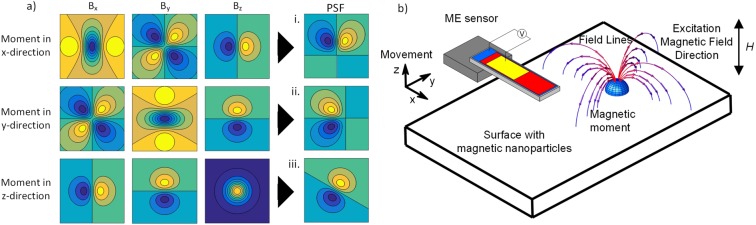
(**a**) Magnetic field components for different orientations of the dipole moment, that are projected onto the sensitive axis of the sensor. This yields the PSF to describe the system. The yellow to green color corresponds to a phase of 0 whereas blue means a phase of *π*. The color bar is different for the different images. The importance lies in the shape of the dipole fields. (**b**) The orientation of the sample surface to the ME sensor that corresponds to the PSF iii. in a).

The integral here is defined over an area *A* with the plane coordinates *a*_1_ and *a*_2_. Because the sensor has a vectorial characteristic, meaning that there exists a sensitive axis, the projection of the field amplitude in the plane of the sensor onto the sensitive axis leads to several possible forms of the PSF as shown in Fig. 4.

The orientation of the sensitive axis, with respect to the longitudinal axis of the sensor, depends on the fabrication^20,22^. In our case, the orientation of the sensitive axis is about 30° to the longitudinal direction in the plane of the sensor. It is important to note that due to the usage of the shape anisotropy, we are left with dipole fields that are antisymmetric with respect to one axis or two axes, because the symmetric fields are attenuated. Thus, the resulting PSF is comprised of the antisymmetric parts of the dipole field. That way, we can only resolve gradients of particle distributions. The PSFs are dependent on the distance and orientation to the sample surface. This is important as the forward modeling is described by a convolution and the smoother the PSF, the stronger larger spatial frequencies of the particle distribution will be attenuated. The inverse process, the deconvolution, then amplifies large spatial frequencies. But higher spatial frequencies are distorted by noise. That way, we want to have a more localized, less smooth, PSF and want to be close to the sample surface. For spatial reasons, and to account for the sensor dimensions, a setup was chosen with the PSF as shown in Fig. 4a)iii. The corresponding orientation to the sample surface is shown in Fig. 4b).

## Experiment

**Figure 5:**
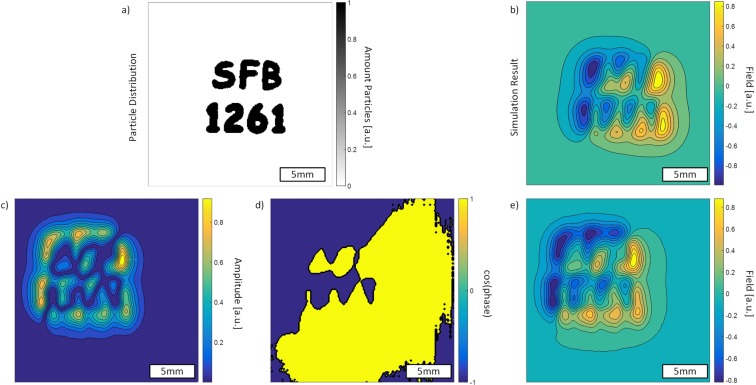
Measurement of a reference sample with the labeling ‘SFB 1261’, the number of our Collaborative Research Center from which this work was funded (see below). (**a**) The approximate reference sample, (**b**) calculated resultant magnetic field from the given distribution, (**c**) measured spatial amplitude, (**d**) measured phase, (**e**) phase and amplitude measurements combined.

We use two sets of Helmholtz coils for the magnetic excitation and for a signal compensation for the sensor. The sensor and the sample are placed in a pair of Helmholtz coils. The magnetic content in the sample corresponds to about 200 µl of an MNP ferrofluid (fluidMAG-CT, particle concentration of 25g/ml, Chemicell, Berlin). This large amount is simply used to ensure large signal amplitudes. We use linear translation stages for the positioning of the sensor and a reference containing MNPs. The sketch of the reference can be seen in Fig. 5a). The letters have an indentation depth of 0.5 mm. In the reference, there were inhomogeneities in the distribution of particles in the letters, unlike shown in the sketch. The reference is moved with respect to the sensor such that the sensor does not misalign with the applied field and stops attenuating, because it is very sensitive to movement. The reference is then moved to sample the magnetic field phase and amplitude at the mechanical resonance of the sensor at equidistant points at a distance to the sensor of about 1 mm. The resulting PSF can then be used to make a prediction for the shape of the measurable magnetic field of the particle distribution [see Fig. 5b)]. The excitation signal is created by an RME FireFace UC at about 2.5 kHz with a sampling frequency of 192 kHz, amplified by an audio amplifier PAS2002 and then connected to the Helmholtz coils. Here we have harmonic distortions of the amplifier at the resonance frequency of the sensor. To accommodate for that, the sensor is aligned to attenuate the signal due to its shape anisotropy (about 4 orders of magnitude for the third harmonic excitation) and furthermore, a cancellation field from the second pair of Helmholtz coils is applied that destructively interferes with the signal at the resonance frequency, such that we cancel the signal down to the noise level. If this is not exactly achieved, the measurements will show a field offset in addition to the noise. We thus reduced the influence of the excitation signal in the third harmonic to only give signals from the nonlinear behavior of the particles. A time signal is amplified via a charge amplifier and measured with the FireFace UC. The signal is sampled at a rate of 32 kHz and the overall time signal length is 4096 samples, to give a spectral resolution of 7.8 Hz. We then use the FFT of that signal and get the amplitude and phase of the signal at the resonance frequency for each measurement position. The measurement time for each position is 2 seconds, though this is related to the measurement script and could altogether be below 1 second, considering the movement time for the stage motors. The amplitude and phase information can then be used to assign a sign to the measurement positions, leading to “positive” and “negative” fields. This is shown in Fig. 5c)–e). We further measured the magnetic fields of two different cell distributions. The preparation of the cell samples is explained in the preparation section below.

## Inverse Problem

Because the reconstruction is very sensitive to noise, we need to regularize the solution for the spatial particle distribution. Furthermore, a restriction for the inverse solution is that the values of the particle distribution are positive. Since the magnetic field sensor is magnetic itself, the influence of the sensor geometry may be nonlinear and thus a description of the sensor as a spatial filter, which averages an inhomogeneous magnetic field, may fail. This may be important when the sensor experiences large inhomogeneities of the magnetic field of the particles. We thus do not incorporate the sensor geometry, which would alter the PSF of the model, and fit the measured data with the modeled PSF.

The discrete model for the generation of the data via convolution [cf. Eq. (9)] of the source distribution with the point spread function $\Psi (i,j)$ is given by

9 $$y(i,j)=\sum _{k=1}^{N}\sum _{l=1}^{M}\Psi (i-k,j-l)x(k,l)+\varepsilon (i,j),$$

where *x*(*k*, *l*), *k* = 1, …, *N*, *l* = 1, …, *M* denotes the original source distribution, within a preselected rectangular region. The term *ε*(*i*, *j*) describes an error term for each measurement position. In this paper, we assume the case *N* = *M*, i.e., the source space is given by a square region, and we furthermore assume that the set of recording positions closely corresponds to the discretization of the source space, such that *i* = 1, …, *N*, *j* = 1, …, *N*.

If $\Psi (i,j)$ is predefined for the same set of grid points as *x*(*k*, *l*) (assuming that it essentially vanishes outside of this region), the sums in equation (9) will access points outside of the preselected region for the source distribution. For this reason, it is necessary to define boundary conditions for *x*(*k*, *l*). There are three commonly employed boundary conditions for this kind of spatial inversion problems: zero boundary condition, periodic boundary condition and reflexive boundary condition. In this paper, we have chosen to employ exclusively the zero boundary condition (assuming that the source distribution vanishes outside of the preselected region), since it agrees with our prior knowledge of the experimental situation.

By (column-wise) vectorizing the matrices *x*(*k*, *l*), *y*(*i*, *j*) and *ε*(*i*, *j*) we can transform this expression into a linear matrix-vector equation,

10 $${\boldsymbol{y}}={\rm{A}}{\boldsymbol{x}}+{\boldsymbol{\varepsilon }},$$

where A denotes the matrix resulting from the vectorization of $\Psi (i,j)$. The dimension of A is *N*^2^ × *N*^2^.

Solving equation (10) for the unknown source distribution ***x*** corresponds to solving the following minimization problem:

11 $$\mathop{{\rm{\min }}}\limits_{{\boldsymbol{x}}}{|{\rm{A}}{\boldsymbol{x}}-{\boldsymbol{y}}|}^{2}.$$

There are analytic approaches available for solving this minimization problem, such as forming the Moore-Penrose pseudoinverse:

12 $$\hat{{\boldsymbol{x}}}={({{\rm{A}}}^{{\rm{T}}}{\rm{A}})}^{-1}{{\rm{A}}}^{{\rm{T}}}{\boldsymbol{y}}.$$

In order to improve the numerical stability of the solution, because the solution is very sensitive to noise in the measured data, the minimization problem of equation (11) should be replaced by the following problem including an additional regularization term:

13 $$\mathop{{\rm{\min }}}\limits_{{\boldsymbol{x}}}({|{\rm{A}}{\boldsymbol{x}}-{\boldsymbol{y}}|}^{2}+{\lambda }^{2}{|{\boldsymbol{x}}|}^{2}).$$

Here, *λ* denotes the regularization parameter. The analytic solution of this problem is given by

14 $$\hat{{\boldsymbol{x}}}={({{\rm{A}}}^{{\rm{T}}}{\rm{A}}+{{\rm{\lambda }}I}_{{N}^{2}})}^{-1}{{\rm{A}}}^{{\rm{T}}}{\boldsymbol{y}},$$

where **I**_*N*_^2^ denotes the *N*^2^-dimensional unity matrix. Let **0**_*N*_^2^ denote the *N*^2^-dimensional column vector of zeros. Then, by defining

15 $$\begin{array}{cc}\mathop{{\rm{A}}}\limits^{\sim }:\,=(\begin{array}{c}{\rm{A}}\\ {\lambda I}_{{N}^{2}}\end{array}), & \mathop{{\boldsymbol{y}}}\limits^{ \sim }:\,=(\begin{array}{c}{\boldsymbol{y}}\\ {{\bf{0}}}_{{N}^{2}}\end{array})\end{array},$$

the problem (13) can be transformed back into the shape of problem (11), with (A,***y***) replaced by $(\tilde{{\rm{A}}},\tilde{{\boldsymbol{y}}})$.

However, the problem we are facing in the present paper, is more difficult than problems (11) and (13), since the estimated source distribution $\hat{{\boldsymbol{x}}}$ must not be negative at any position, *i*.*e*., a set of inequality constraints applies:

16 $${x}_{i}\ge 0\,,\,i=1,\ldots ,{N}^{2}.$$

The corresponding constrained minimization problem cannot be solved by analytic expressions like equations (12) and (14). Instead, iterative numerical algorithms are required. The problem is known as *non-negative least-squares* (NNLS), and a considerable number of algorithms have been proposed for its solution, beginning with the classical NNLS algorithm from Lawson & Hanson^23^. After exploring various available algorithms, we have decided to employ a particular gradient descent algorithm, known as *subspace Barzilai-Borwein non-negative least-squares* (SBB-NNLS) algorithm^24^.

Prior to solving a non-negative least-squares problem, a value for the regularization parameter needs to be chosen. Again, there are algorithms available for this purpose, such as the L-curve method or generalized cross-validation^25^. In practice, suitable values for *λ*^2^ may also be obtained simply by visual inspection of the resulting solutions.

**Figure 6:**
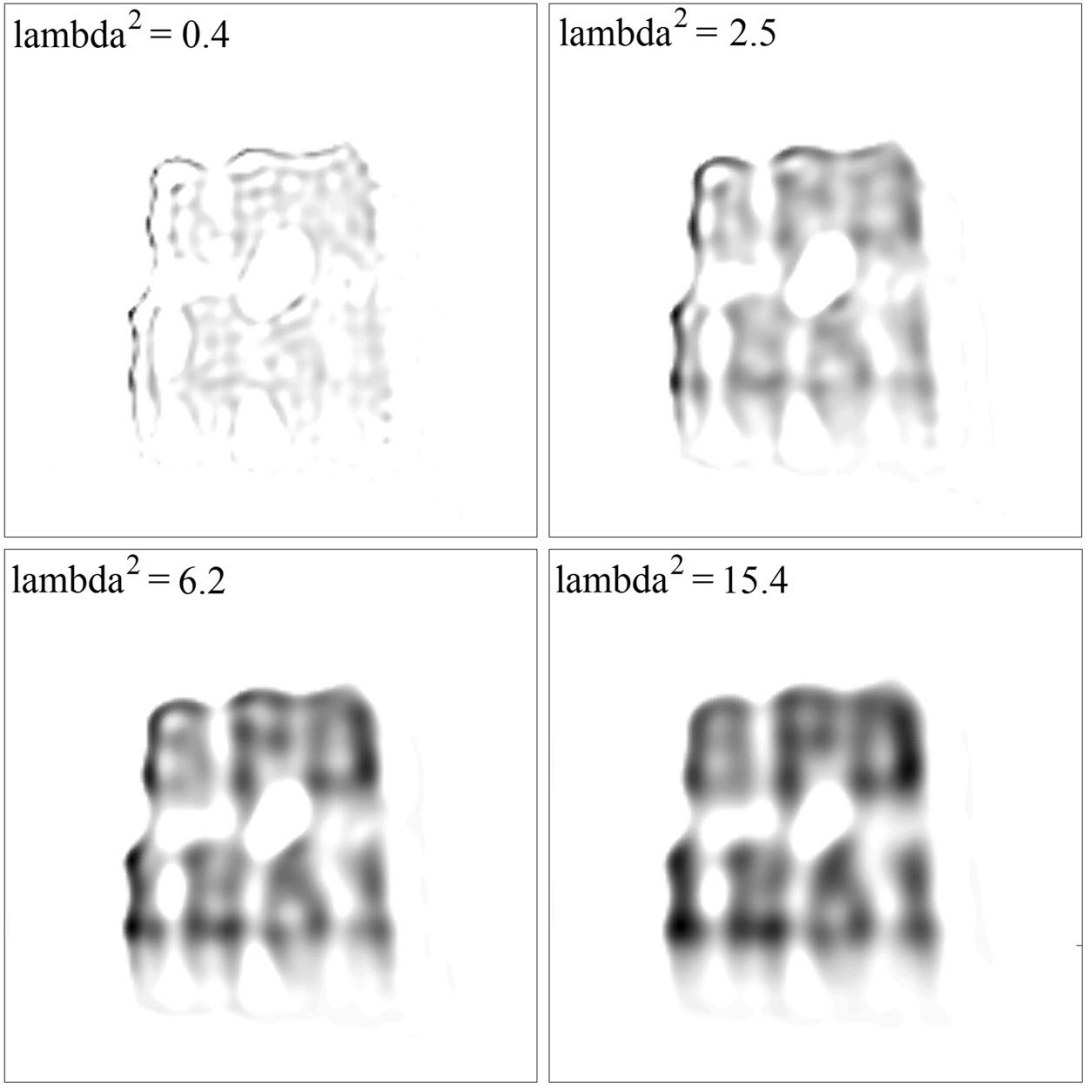
Estimated source distributions for magnetic field data recorded by an ME sensor from an experimental distribution of particles forming the letters ‘SFB 1261’. The data were processed by the SBB-NNLS algorithm with regularization. For the regularization parameter, four different values were chosen, as inserted. Densities of estimated source distributions are given by a black-white color map, with white corresponding to zero and black to the maximum density (maximum density differs for different regularization parameters).

Results of applying the SBB-NNLS algorithm to MPM data for four different values of *λ* are shown in Fig. 6. The data were obtained from a distribution of particles prepared such that it forms the letters ‘SFB 1261’ (see Fig. 5). The size of the discretized source space was *N*^2^ = 153^2^. From the figure it can be seen that for small values of *λ*^2^ the solution is dominated by ripple-like structures, while for large values it results in a highly blurred solution. The transition from the ripple-like to the blurred solutions occurs somewhere close to *λ*^2^ ≈ 6. We see that the reconstructed solution is still smeared out instead of located in the letters. This blurring cannot necessarily be only attributed to the noise in the measurement, but also to the relatively large dimensions of the sensor itself with respect to the letters in the pattern. We thus only fit an approximate model to the data.

**Figure 7:**
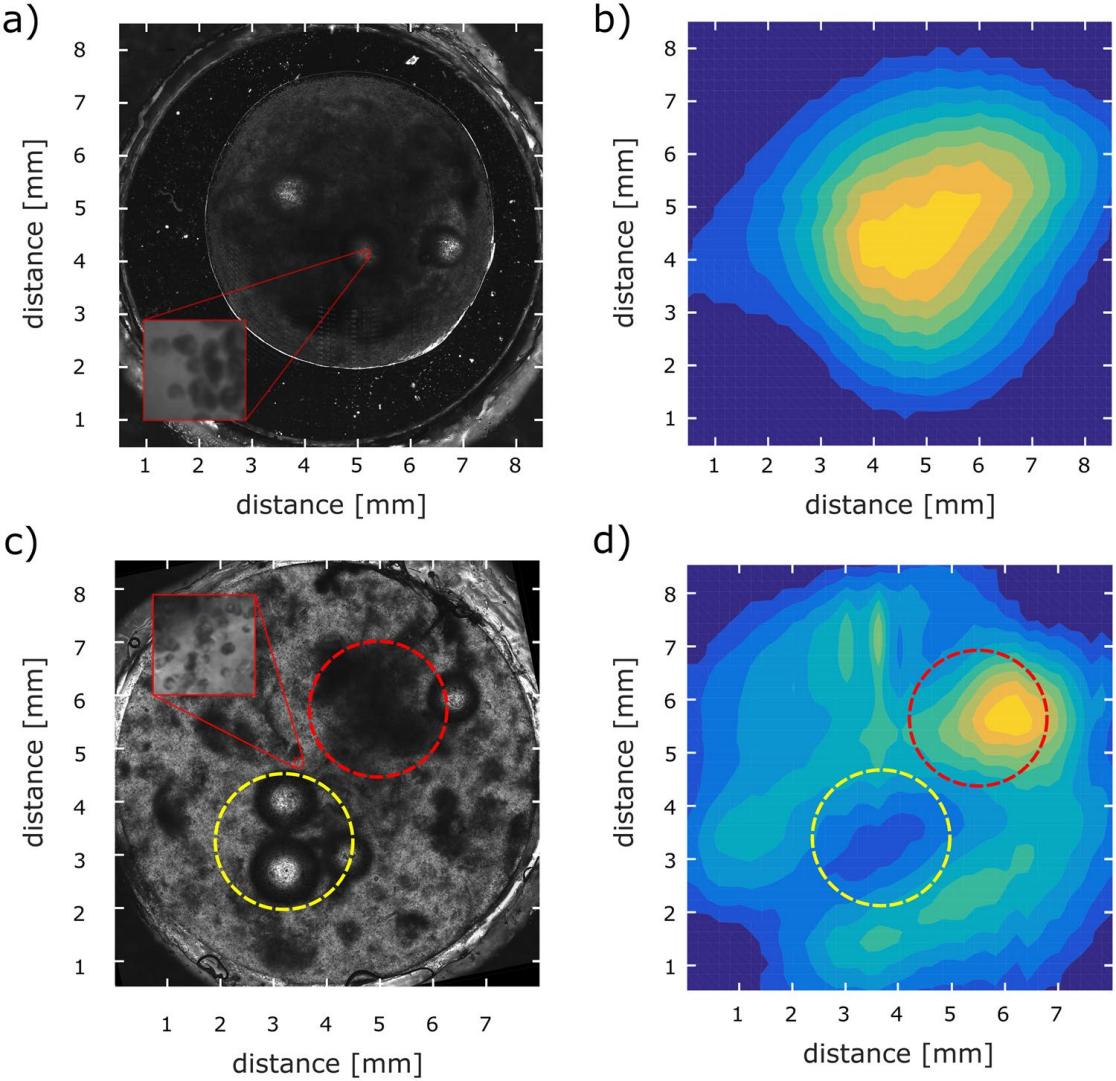
(**a**) Light microscope image of clustered magnetically labeled cells. The inset shows a close up of the cell distribution. (**b**) Reconstructed cell distribution from magnetic measurements. (**c**) Light microscope image of a smeared out cell droplet with magnetically labeled cells. The square inset shows a close up of the cell distribution. (**d**) Reconstructed cell distribution from magnetic measurements.

We further investigated two distributions of magnetically labeled cells embedded into a Matrigel scaffold in two cylindrical, 1 mm deep cavities. Each of the cavities contains approximately 8×10^5^ cells. Light microscopy images of the cells inside the sample holders are shown in Fig. 7 on the left. The cell distribution reconstructed from the measurement of the magnetic field is shown in the same figure on the right. We investigated two cases: In the first case [Fig. 7a,b)], cells were clustered in a small area of the sample holder, whereas in the second case [Fig. 7c,d)] cells were distributed throughout the sample holder. In both cases the cell distribution reconstructed by MPM matches very well with the optically observed distribution. In the second case [Fig. 7c)] a region with a high cell concentration is highlighted both in the microscopic image and in the reconstruction [Fig. 7d)] with a red circle. Another region with a low cell concentration is in both images highlighted with a yellow circle. The cell concentration in this region is low because of air bubbles in the Matrigel scaffold.

## Discussion

We have presented a new measurement approach to detect signals from MNPs using ME sensors. We used the nonlinear response of MNPs to map a magnetic field of a reference sample containing particles in a pattern. We modeled the forward problem and, on that basis, we solved the inverse problem using an L2 penalization with a nonnegativity constraint. Using this approach, we showed that it is possible to detect magnetically labeled cells and compared light microscope images with reconstructed measurements.

In the following, considerations and comparisons of this approach will be discussed in the context of an imaging modality with respect to quantification and spatial resolution. Subsequently, future perspectives and possible improvements will be elucidated.

The spatial resolution of the presented approach depends on the noise level in the measurements and the accuracy of the model. Since the model does not include the geometry of the sensor itself and how an inhomogeneous field is averaged over the sensor plane, we only fit an approximate model to our data. This will ultimately determine the amount of detail that can be resolved in the distribution, if this contribution is not included in the model. Furthermore, the need for regularization to prevent overfitting implies a less detailed solution. The source discretization for the inverse problem does not necessarily need to be of the same dimensions as the number of measurements. If we have an overdetermined system with fewer pixels (in 2D, or voxels in 3D respectively), we should still be able to accurately determine the magnetic content in a given region, because the model matrix is less ill conditioned.

For a 3D reconstruction of MNP distributions using the introduced approach, there will be a bias of the regularized solution to the sources near to the sensors^26^. To resolve deeper lying sources of the MNP distributions, a larger amount of magnetic content is beneficial, or the source should be discretized more coarsely. Overall, the depth resolution of this approach can be expected to be poor in comparison to techniques such as MRI or MPI. MRX imaging systems can use local excitation coils and the sequential activation can be used to reconstruct sources in different positions of a 3D sample more accurately^10^. This is a benefit of using inhomogeneous magnetic fields. Such approaches may also be used in the future with ME sensors. Another important issue is the duration of the measurements. The overall measurement time could be greatly decreased by using ME sensor arrays. This will be investigated in the future.

The approach to solve the inverse problem can also be further investigated. Here, we used SBB-NNLS with an L2 penalty on the solution vector to reconstruct the solution and include constraints. Nonetheless, depending on the source distribution and whether we want to preserve edges or want a more sparse solution, regularization techniques with an L1 penalty such as total variation could be used and may enhance the reconstruction. Another possible enhancement would be the measurement of several projections of the magnetic field of the particles for the reconstruction.

The measurements of the magnetic fields of the magnetically labeled cells could be further enhanced by using a larger excitation amplitude or using larger MNPs, to harvest more of the nonlinearity of the MNPs. This will ultimately limit the resolution of the inverse solution. Another possibility is to label the cells with more MNPs. Here we were able to measure the distribution of magnetically labeled cells with overall 8×10^5^ cells at 1 mm distance with a pixel size of 0.25×0.25 mm^2^.

Considering the ratio of the maximum value of the magnetic field in the cell measurement to the field that is measured when no sample is present and that the measurement signal scales linearly with the amount of magnetically labeled cells, we can estimate a detection limit at 1 mm distance to about 2000 cells. Though it is important to note that for a proper reconstruction a good signal-to-noise ratio is needed. Furthermore, since the magnetic dipole field decays with distance cubed, this detection limit will as well decay in the same manner. We expect to achieve at least one order of magnitude improvement in the signal strength by employing the approaches outlined above. In a study by Zheng *et* *al*.^6^ the detection limit for magnetically labeled cells using their MPI setup was estimated to be 200 cells. The presented MPM approach may be comparable to MRX imaging in terms of quantification in the future.

## Materials and Methods

### Preparation of cell samples

Rat embryonic fibroblasts (Ref52 wt) were grown in 6-well plates (Sarstedt AG & Co.) using Dulbecco’s modified Eagle’s medium (DMEM, Biochrom) supplemented with 10% fetal bovine serum (Biochrom) at 37 °C, 5% CO2, and about 90% humidity. Once confluent, superparamagnetic nanoparticles (50 µg/ml) were added to 3 ml medium for 24 h. The nanoparticles have a hydrodynamic diameter of 50 nm and the magnetic core is embedded in a polymer matrix consisting of citric acid (fluidMAG-CT, Chemicell, Berlin). Previous magnetic measurements of cells suggest about 10 pg Iron per cell.

After 24 h the adherent cells are washed with PBS three times to remove all nanoparticles from the supernatant. Subsequently, the cells are trypsinised and spinned down. The supernatant is removed carefully, and the cells are resuspended into 100 µl of Matrigel (Matrigel Matrix Phenol Red Free 10 ml, Corning).

In the experiments we use two sample holders that consist of a 1 mm thick PMMA slice, each with a cavity of 8 mm diameter in the center. Into each of these cavities a droplet of 30 µl cell-Matrigel solution is added. To stabilize the droplet, the remaining volume of the hole is filled with pure Matrigel. In one of the two samples the cell droplet is mixed with the surrounding Matrigel, so that the cells are distributed over a much larger area. Both samples contain approximately 8×10^5^ cells.

### Sensor Fabrication

The ME-sensor has the shape of a cantilever with 3 mm length and 1 mm width and consists of 50 µm poly-silicon with the magnetostrictive and piezoelectric phase on top. It is fabricated by MEMS technology from an oxidized silicon/poly-silicon wafer. Effectively 1 µm of the highly magnetostrictive alloy Fe_70.2_Co_7.8_Si_12_B_10_ (FeCoSiB) is sputtered in the form of an exchange bias stack with (Ta-5nm/Cu-3nm/MnIr-8nm/FeCoSiB-200nm) × 5. On top of this, 2 µm highly piezoelectric aluminium nitride is sputtered using a low temperature deposition process as described in ref.^27^ with 5 nm Ta and 100 nm Pt as seed. The cantilever is released using wet and dry etching technologies. Details of the process can be found in ref.^28^. The piezoelectric voltage generated by the magnetostrictive induced bending of the cantilever is read out by a plate capacitor using the Pt seed and a golden top electrode. A silicon frame of 7.5 mm × 11 mm surrounds the sensor for easy handling. The sensor is annealed in an oil bath for 30 min at 250 °C with a magnetic field of 1 kG applied 60° to the long axis of the cantilever. By this, the exchange bias effect imposed, which shifts the magnetostriction curve in such a way, that the highest slope is at zero magnetic field. Details to this process can be found in ref.^21^.

## Supplementary information

Voltage noise at amplifier output

## Data Availability

The data that support the findings of this study are available from the corresponding author upon request.
