# Supplementary material for: Magnetic particle mapping using magnetoelectric sensors as an imaging modality
Source: Sci Rep. 2019 Feb 14;9:2086. doi: 10.1038/s41598-018-38451-0 (PMC6375992; doi:10.1038/s41598-018-38451-0)
Supplement: Supplementary file 1 — Voltage noise at amplifier output [file 41598_2018_38451_MOESM1_ESM.pdf]

Magnetic particle mapping using magnetoelectric sensors as an imaging modality

Ron-Marco Friedrich[1], Sebastian Zabel[1], Andreas Galka[2], Nils Lukat[3], Jan-Martin Wagner[1,4], Christine Kirchhof[5], Eckhard Quandt[5], Jeffrey McCord[6], Christine Selhuber-Unkel[3], Michael Siniatchkin[2] & Franz Faupel[1]**

[1]Christian-Albrechts-Universität zu Kiel, Institute for Materials Science, Chair for Multicomponent Materials, Kiel, 24143, Germany

[2]Christian-Albrechts-Universität zu Kiel, Institute for Medical Psychology and Medical Sociology, Kiel, 24105, Germany

[3]Christian-Albrechts-Universität zu Kiel, Institute for Materials Science, Biocompatible Nanomaterials, Kiel, 24143, Germany

[4]Christian-Albrechts-Universität zu Kiel, Institute for Materials Science, Functional Nanomaterials, Kiel, 24143, Germany

[5]Christian-Albrechts-Universität zu Kiel, Institute for Materials Science, Inorganic Functional Materials, Kiel, 24143, Germany

[6]Christian-Albrechts-Universität zu Kiel, Institute for Materials Science, Nanoscale Magnetic Materials and Magnetic Domains, Kiel, 24143, Germany

*rmfr@tf.uni-kiel.de, ff@tf.uni-kiel.de

Supplementary

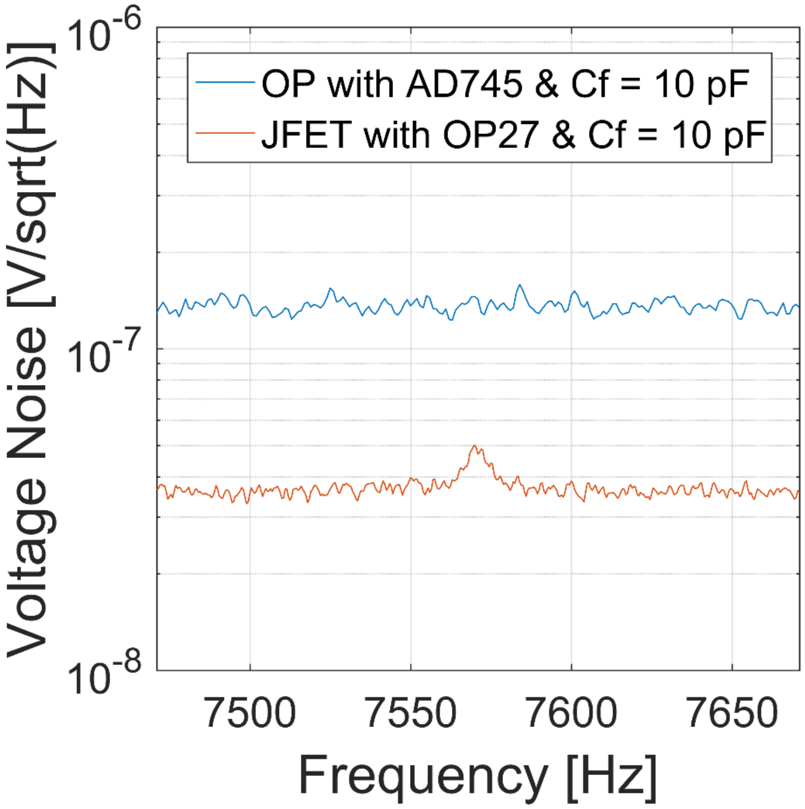

Noise spectra of the sensor, which was used in this paper. The blue curve is the noise spectrum using the charge amplifier (using AD7 45), which was used for the measurements. The red curve shows the noise spectrum using an enhanced charge amplifier (with JFET), which shows the thermal mechanical noise at the resonance of the sensor.
